# Nano-Organization at the Synapse: Segregation of Distinct Forms of Neurotransmission

**DOI:** 10.3389/fnsyn.2021.796498

**Published:** 2021-12-22

**Authors:** Natalie J. Guzikowski, Ege T. Kavalali

**Affiliations:** ^1^Department of Pharmacology, Vanderbilt University, Nashville, TN, United States; ^2^Vanderbilt Brain Institute, Vanderbilt University, Nashville, TN, United States

**Keywords:** nanocolumn, spontaneous neurotransmission, asynchronous neurotransmission, synchronous neurotransmission, synaptic transmission and plasticity

## Abstract

Synapses maintain synchronous, asynchronous, and spontaneous modes of neurotransmission through distinct molecular and biochemical pathways. Traditionally a single synapse was assumed to have a homogeneous organization of molecular components both at the active zone and post-synaptically. However, recent advancements in experimental tools and the further elucidation of the physiological significance of distinct forms of release have challenged this notion. In comparison to rapid evoked release, the physiological significance of both spontaneous and asynchronous neurotransmission has only recently been considered in parallel with synaptic structural organization. Active zone nanostructure aligns with postsynaptic nanostructure creating a precise trans-synaptic alignment of release sites and receptors shaping synaptic efficacy, determining neurotransmission reliability, and tuning plasticity. This review will discuss how studies delineating synaptic nanostructure create a picture of a molecularly heterogeneous active zone tuned to distinct forms of release that may dictate diverse synaptic functional outputs.

## Introduction—Classical View of Single Active Zone Synapses

In the 1960s the canonical synaptic transmission pathway was established; neurotransmission is initiated by an action potential arriving at the presynaptic terminal, presynaptic calcium influx is triggered, synaptic vesicles fuse with the presynaptic membrane and ultimately release neurotransmitter (Katz, 1969; Südhof, 2013). This seminal work set the stage for the investigation of neurotransmission at numerous synapse types across the nervous system in diverse animal models with electrophysiology. This original view of synaptic transmission holds true today albeit with knowledge of the specificity, segregation, and molecular mechanisms that mediate and regulate neurotransmission. Today, three different modes of neurotransmission are categorized by their time scale relative to a stimulus as well as calcium dependence. Action potential dependent release is composed of synchronous and asynchronous phases, where synchronous release strictly adheres to the timing of incoming presynaptic action potentials and asynchronous release is only loosely coupled to stimulation temporally. In contrast, spontaneous release happens independently of action potentials in a quantal manner, where single synaptic vesicles fuse and release neurotransmitter in a quasi-random fashion (Kavalali, 2015). Studies conducted at the *Drosophila* neuromuscular junction as well as hippocampal synapses have demonstrated that a single active zone is capable of synchronous, asynchronous, and spontaneous release while some synapses exclusively execute spontaneous or evoked neurotransmission, creating a dynamic neuronal network dependent on distinct forms of neurotransmission (Atasoy et al., 2008; Melom et al., 2013; Peled et al., 2014; Reese and Kavalali, 2016). Each of the three modes of neurotransmission rely on distinct molecular frameworks to ultimately accomplish complex information processing (Kononenko and Haucke, 2015; Chanaday and Kavalali, 2017).

Recently, our understanding of neurotransmission has expanded to include both action potential dependent release and spontaneous release which have distinct physiological roles governed by a molecularly heterogeneous active zone (Andreae and Burrone, 2018; Gonzalez-Islas et al., 2018; Kavalali, 2018). The expansion of our synaptic “world-view” to include the nuances of different modes of neurotransmission relays the importance of the molecular pathways and synaptic structures that support release and presents broader implications for learning, memory, as well as associated disease processes. With the continued advancement of tools to probe release segregation, the notion that neurotransmission occurs via distinct pathways has been bolstered. How this segregation of release is maintained by the nano-organization of the synapse to transduce complex information processing will be the focus of this review.

### Synaptic Efficacy

The fundamental reason we study the synapse is to understand how one neuron influences its targets. Therefore, to elucidate this process, we must consider how synaptic efficacy is shaped by multiple molecular pathways and different modes of neurotransmission. Synaptic efficacy defined as the ability of a presynaptic input to influence a postsynaptic response is classically considered within the context of action potential induced presynaptic calcium signaling and subsequent neurotransmitter release. The synaptic efficacy of an active zone or release site is traditionally determined by the release probability, the probability that upon arrival of an action potential to the synaptic terminal a synaptic vesicle will fuse and there will be subsequent neurotransmitter release. The size of the readily releasable pool, which includes vesicles docked at the active zone that fuse first in response to stimulation, and the fusion propensity of each synaptic vesicle dictates this release probability and ultimately synaptic strength (Rey et al., 2015; Chanaday and Kavalali, 2018). This view of synaptic strength and synaptic vesicle organization focused solely on evoked neurotransmission assumes a rather homogeneous synaptic vesicle pool and equally homogeneous organization of the active zone. In addition, it excludes other modes of neurotransmission and how their distinct functional roles may shape, guide, or determine synaptic efficacy.

## Molecular Mechanisms of Distinct Forms of Release

**FIGURE 1:**
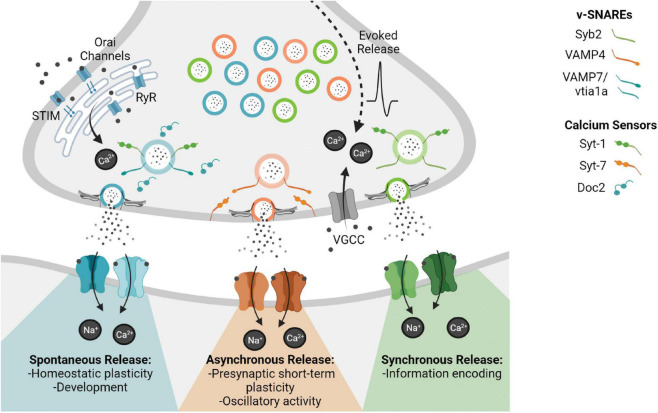
This figure depicts the different modes of neurotransmission and their distinct downstream signaling pathways. Synchronous release transmits precise timing information across the synapse enabling information transfer with fidelity, whereas asynchronous release is thought to regulate short term plasticity and oscillatory activity. In contrast, spontaneous release is action potential independent although it may rely on alternative calcium sources, such as endoplasmic reticulum (ER) mediated calcium via ryanodine receptors (RyR) or store-operated calcium entry via Orai channels activated by the ER calcium sensor STIM. Spontaneous release is thought to play a role in synapse development as well as homeostatic synaptic scaling shaping synaptic efficacy.

Historically the organization of synaptic vesicles within the presynaptic terminal was defined by their propensity to fuse in response to stimulation and ultrastructural localization. Thus, creating a pool organization of synaptic vesicles comprised of the readily releasable pool, vesicles that fuse first in response to stimulation, and the reserve pool, the pool that replenishes the readily releasable pool, which shows limited synaptic vesicle trafficking during physiological activity (Alabi and Tsien, 2012). However, this pool organization does not fully account for the dynamics of release with regard to different modes of neurotransmission. Understanding the heterogeneity of synaptic vesicle molecular composition and how this heterogeneity dictates release and trafficking creates a more accurate classification system of synaptic vesicle organization in the presynaptic terminal. The diversity in synaptic vesicle molecular composition of v-SNAREs (vesicle-soluble *N*-ethylmaleimide-sensitive factor (NSF) attachment protein receptor) and calcium sensors distinguishes synaptic vesicle populations indicating that unique molecular compositions may designate synaptic vesicles for different forms of release (Figure 1) (Crawford and Kavalali, 2015). Furthermore, there is increasing evidence that different calcium sources mediate distinct modes of release (Kavalali, 2020).

### Synaptic Vesicle Associated SNAREs, v-SNAREs

The canonical SNARE complex includes synaptic vesicle associated protein Synaptobrevin 2 (Syb2, also called VAMP2) that forms a complex with the plasma membrane SNAREs synaptosomal-associated protein 25 (SNAP25) and syntaxin 1 to drive rapid action potential-evoked synaptic vesicle fusion (Südhof and Rothman, 2009). Early experiments investigating the selective loss of canonical SNAREs induced large effects on evoked release while spontaneous release was maintained. The genetic deletion of Syb2 in mice caused the loss of calcium dependent evoked release while residual spontaneous release and to some extent asynchronous release were still present (Schoch et al., 2001; Deák et al., 2004). This initial work stimulated further research into which SNAREs are mediating different modes of neurotransmission as molecular perturbations selectively effected spontaneous and evoked release in distinguishable ways.

Fluorescence imaging experiments in conjunction with electrophysiology have shown that Vps10p-tail-interactor-1a (vti1a) containing vesicles traffic in the absence of activity and vesicle-associated membrane protein 7 (VAMP7) containing vesicles also preferentially traffic in response to resting calcium signals, thus both v-SNAREs specifically drive spontaneous release (Hua et al., 2011; Ramirez et al., 2012; Bal et al., 2013; to see a full list of SNAREs associated with the synaptic vesicle proteome; see Takamori et al., 2006). Furthermore, despite both synchronous and asynchronous release being action potential dependent, vesicle-associated membrane protein 4 (VAMP4) selectively maintains asynchronous release demonstrated by differential trafficking with minimal Syb2 trafficking overlap (Raingo et al., 2012). In addition to the functional evidence that these alternative v-SNAREs maintain distinct trafficking pathways, biochemical evidence is consistent with their distinct functions. As for instance VAMP4 forms stable SNARE complexes independent of Syb2 and these complexes do not interact with synaptotagmin-1 and complexins, two protein that are essential for rapid synchronous evoked release (Raingo et al., 2012). The elucidation of specific v-SNAREs that selectively maintain different modes of release reveals a synaptic terminal composed of subpopulations of vesicles defined by their heterogeneous distribution of molecular components (Ramirez et al., 2012; Revelo et al., 2014; Walter et al., 2014). Alternative v-SNAREs act as synaptic vesicle molecular tags for the organization of synaptic vesicles into pools, conferring distinct release properties, downstream functional roles, and the selective regulation of neurotransmitter release (Figure 1) (Mori et al., 2021).

### Calcium Sensors

Differences in the molecular machinery to maintain synchronous, asynchronous, and spontaneous release extend beyond SNARE machinery and include calcium sensing proteins. The diversity in calcium sensitivity between synchronous, asynchronous, and spontaneous release is complex; with differential degrees of absolute dependence, different sources of calcium mediating release, as well as differential calcium dependences between excitatory and inhibitory spontaneous release (Courtney et al., 2018; Williams and Smith, 2018; Lin et al., 2020).

Synaptotagmins are a family of calcium sensing proteins implicated in synaptic vesicle exocytosis. Synaptotagmin-1 (Syt-1) is required for synchronous release mediating the calcium sensitivity of fast evoked neurotransmitter release. While the loss of Syt-1 impairs synchronous release, it unclamps spontaneous release and augments asynchronous release, regulating release bidirectionally (Maximov and Südhof, 2005; Liu et al., 2009). Syt-1 is a low affinity calcium isoform making it only a reliable calcium sensor following voltage gated calcium channel (VGCC) opening and subsequent nanodomain elevation of calcium. Whereas Synaptotagmin-7 (Syt-7), the proposed asynchronous calcium sensor, has a 10-fold higher calcium affinity than Syt-1, giving it utility on a longer timescale post VGCC opening. Syt-7 loss of function mutations drastically reduce asynchronous release and associated synaptic vesicle trafficking providing the molecular framework for the differential timing of these two types of action potential dependent release (Geppert et al., 1994; Sugita et al., 2002; Bacaj et al., 2013; Li et al., 2017).

Although the precise nature of calcium sensitivity of spontaneous release is still a matter of debate, soluble calcium sensors of the Doc2-like protein family are thought to regulate spontaneous release in addition to synaptotagmin (Groffen et al., 2010; Pang et al., 2011). A quadruple knockdown strategy to eliminate members of the Doc2 family caused a reduction in spontaneous neurotransmission while action potential-evoked neurotransmission remained relatively normal. This protein loss also caused a subsequent increase in synaptic strength, suggesting that spontaneous neurotransmission is able to communicate independently with the postsynaptic neuron and trigger downstream signaling cascades that regulate the synaptic state (Ramirez et al., 2017). This regulation of release via calcium dynamics shows even more complexity with excitatory spontaneous signaling preferentially regulated by Doc2α and inhibitory spontaneous signaling by both Doc2β and Syt-1 (Courtney et al., 2018).

## Functional Consequences of Distinct Forms of Release

The investigation into molecular components that drive different modes of release implies there are distinct functional consequences for synchronous, asynchronous, and spontaneous release. These studies have addressed why different modes of release are maintained in separable pathways and how they contribute to the physiological function of the synapse. In particular, asynchronous and spontaneous forms of release are poorly understood in comparison to evoked synchronous release, however, recent work has identified dedicated functional roles that are unique and specific to each mode of release.

## Spontaneous Release

Spontaneous release events were originally viewed as random errors at the synaptic terminal deviating from canonical action potential dependent release, and described as biological noise (Fatt and Katz, 1950). However recent evidence suggests that spontaneous release has a specialized role in both regulating synapse and circuit development in addition to the maintenance of synapse dynamics in several organisms (Huntwork and Littleton, 2007; Choi et al., 2014; Andreae and Burrone, 2015; Banerjee et al., 2021). Furthermore recent findings on the role of aberrant spontaneous neurotransmission in neurological disease as well as ketamine’s rapid antidepressant action arising from spontaneous release modulation reflect the utility of different modes of release at the synapse (Autry et al., 2011; Alten et al., 2021). Ultimately revealing spontaneous neurotransmission as an autonomous mode of release involved in a broad range of functions (Kavalali and Monteggia, 2020).

### Development

Early stages of development are characterized by high levels of spontaneous release and resting synaptic vesicle cycling as compared to minimal evoked release (Molnár et al., 2002; Andreae et al., 2012). Previously the importance of spontaneous activity in synapse formation has been speculative due to the lack of a proposed function of developmentally elevated spontaneous release (Andreae and Burrone, 2018). However, key studies at the *Drosophila* neuromuscular junction during development have demonstrated how the frequency of spontaneous release events are directly correlated with synaptic structure. Experimentally facilitating miniature postsynaptic currents (mPSCs) leads to the subsequent increase in presynaptic boutons and the blockade of mPSCs leads to abnormal neuromuscular junctions characterized by reduced growth and surface area (Choi et al., 2014). In addition, spontaneous glutamate release events are thought to regulate dendritic arbors by acting as cues for dendritic outgrowth, a process not modulated by evoked release (Andreae and Burrone, 2015). Spontaneous release maintains these developmental pathways that are not controlled by evoked release delineating spontaneous release specific biochemical pathways (Figure 1; Andreae and Burrone, 2018).

### Homeostatic Plasticity

In response to global changes in neuronal activity synaptic weights are up- or down-scaled to maintain homeostatic set points of neurotransmission. This homeostatic synaptic scaling maintains the relative differences in synaptic weights between synapses on a neuron, vital for information processing, while still allowing the system to adapt to environmental levels of activity (Turrigiano and Nelson, 2004; Kavalali and Monteggia, 2020). Global changes in activity elicited by complete suppression of action potentials (tetrodotoxin treatment) or conversely disinhibition (bicuculine treatment) can regulate this scaling phenomenon by up or down regulation of postsynaptic receptor density. However, synaptic scaling can also be triggered by alterations in action potential independent neurotransmitter release or via direct manipulation of neurotransmission without gross changes in activity levels (Gonzalez-Islas et al., 2018).

Reduction in N-methyl-D-aspartate receptor (NMDAR) mediated miniature excitatory postsynaptic currents (mEPSCs) via postsynaptic manipulations triggers Eukaryotic Elongation Factor 2 Kinase mediated synaptic upscaling via local dendritic translation (Sutton et al., 2006; Aoto et al., 2008; Reese and Kavalali, 2015). Not only is this NMDAR synaptic scaling pathway separable from the canonical NMDAR mediated long term potentiation (LTP) pathway but it is also implicated as a substrate for disease intervention in major depressive disorder (Lin et al., 2018). In parallel the loss of spontaneous specific release machinery, vti1a and VAMP7, triggers synaptic scaling of α-amino-3-hydroxy-5-methyl-4-isoxazolepropionic acid receptors (AMPARs) as reflected by increased mEPSC amplitudes (Crawford et al., 2017). Furthermore, the blockade of spontaneous γ-aminobutyric acid (GABA)-ergic signaling leads to multiplicative downscaling at excitatory synapses via brain-derived neurotrophic factor transcription and signaling, demonstrating an exclusive role of spontaneous release in the relationship between excitatory and inhibitory synapses (Horvath et al., 2021). These specific pre- and post-synaptic perturbations ultimately reveal how spontaneous release functions to maintain basal levels of synaptic efficacy, through separable mechanisms from evoked release (Figure 1).

## Asynchronous Release

### Plasticity

Due to the slow dynamics of asynchronous release–vesicle fusion occurring with 10–100 ms delay after an action potential –it is speculated to impact synaptic plasticity. However, neuron and synapse specific diversity in the degree of asynchronous release make the generalization of the physiological significance of asynchronous release difficult. Syt-7, putative asynchronous calcium sensor, is required for short-term presynaptic facilitation at some synapses (Jackman et al., 2016). While at fast synapses, with minimal asynchronous release, Syt-7 is not required for short-term plasticity but the fidelity of synchronous release allowing for prolonged synaptic signaling (Luo and Südhof, 2017).

### Information Encoding

Fast synchronous release is often thought to be the main mode of information transfer due to its tight temporal coupling with presynaptic action potentials. Nevertheless, loss of synchronous neurotransmission by *in vivo* knockdown of Syt-1 in the hippocampus still allowed the acquisition of fear memories, albeit with impairments, suggesting that asynchronous release detected after Syt-1 loss-of-function is still sufficient to encode the majority of the memory. While this result does not negate the importance of release timing in brain circuits, it indicates that action potential bursts and subsequent asynchronous release can be critical drivers for information encoding (Xu et al., 2012).

### Oscillatory Activity

Understanding the degree of asynchronous release in different neuronal subtypes provides insight into the molecular diversity and input specificity of inhibitory interneurons in the hippocampal circuit. Cholecystokinin (CCK)–expressing GABAergic interneurons (innervate pyramidal cells at soma and proximal dendrites) are characterized by high asynchronous activity as compared to other interneuron subtypes. Therefore their prolonged asynchronous GABA release generates long lasting inhibition that modulates circuit activity (Hefft and Jonas, 2005). Based on CCK–expressing GABAergic interneurons’ role in maintaining low frequency oscillations of the hippocampus, their prominent asynchronous release is suspected to play a key role in generating hippocampal theta rhythms (Klausberger and Somogyi, 2008; Rozov et al., 2019). Delineating the physiological function of asynchronous release demonstrates its unique and specific role creating the context to further study the molecular underpinnings of each mode of release (Figure 1).

## Nanostructure

The molecular diversity of synaptic vesicles as well as distinct functional roles of each mode of release challenge the notion of a molecularly homogeneous organization within the synaptic terminal. The proposed structure of the synapse now incorporates the nano-organization of proteins throughout the synapse, including protein density gradients in the active zone, synaptic cleft, receptor localization, and postsynaptic density creating a physical trans-synaptic alignment of proteins termed a nano-column (Tang et al., 2016). Synaptic nanocolumns are suspected to have a role in facilitating release differentially providing the framework at which the synapse is able to maintain different modes of release via parallel pathways.

## Distinct Postsynaptic Targets

### Excitatory Synapses

The application of use dependent drugs and optical imaging have demonstrated that different modes of neurotransmission target distinct postsynaptic receptors (Atasoy et al., 2008; Melom et al., 2013; Peled et al., 2014). Initial studies at excitatory synapses took advantage of use dependent receptor blockers to probe postsynaptic receptor segregation. The application of use dependent NMDAR blocker, MK-801, demonstrated a near complete segregation of NMDAR response to spontaneous and evoked glutamate release (Atasoy et al., 2008; Reese and Kavalali, 2016). The use of philanthotoxin, use dependent blocker of GluR2 lacking AMPARs, extended this notion to evoked and spontaneous glutamate release dependent activation of distinct sets of AMPARs (Sara et al., 2011; Peled et al., 2014). These results demonstrate the independence of evoked and spontaneous release probabilities as well as spatial segregation of the two forms of release.

### Inhibitory Synapses

Despite accumulating evidence of nano-organization at excitatory synapses, segregation of distinct forms of release at the inhibitory synapse is relatively under-investigated. The distinct functional roles of evoked and spontaneous release at excitatory synapses provides rationale for robust segregation, however, the degree of segregation and functional outcomes of evoked and spontaneous inhibitory signaling are unknown. A recent study has uncovered partial segregation of evoked and spontaneous release at an inhibitory synapse. The utilization of picrotoxin, a use dependent GABA_*A*_R channel blocker, demonstrated that approximately 40% of GABA_*A*_Rs are exclusively activated by evoked release (Horvath et al., 2020).

Historically the main role of inhibitory signaling has been cast as modulating excitatory neurotransmission and regulating a neuron’s propensity to fire action potentials. However, only recently has the biochemical signaling of inhibitory neurotransmission been addressed due to the few known targets of chloride. The discovery that with-no-lysine kinases (WNKs) function as chloride sensors and second messenger cascades downstream of GABAergic chloride current provide insight into the dynamic nature of the inhibitory synapse (Piala et al., 2014; Heubl et al., 2017; Chen et al., 2019). Further adding to the complexity of the inhibitory synapse, GABAergic signaling is excitatory during early development, influencing synapse plasticity and growth to generate functional circuits (Ben-Ari, 2002). Understanding if there are common principles governing nanostructure and the segregation of release at inhibitory synapses as seen at excitatory synapses is crucial, however, it remains unclear if a partial segregation mediates differential signaling of inhibitory spontaneous and evoked neurotransmission and how this segregation is achieved (Horvath et al., 2020).

### Neurotransmitter Receptor Dynamics

The nanocolumn organization discussed above allows for concentrated increases in neurotransmitter at designated regions along the active zone, aligned with specific receptor sub-populations. The significance of transient localized increases in neurotransmitter is bolstered by studies investigating the segregation of release via receptor activation, neurotransmitter receptor dynamics, and synaptic plasticity.

To understand the implications of synaptic nanocolumns, it is first important to establish the essential role of the site of neurotransmitter release and receptor localization in determining the efficacy of neurotransmission. The traditional picture of the synapse assumes a homogeneous structure whereby synaptic strength is governed by the number of receptors in the postsynaptic density and neurotransmitter is released at any place within the active zone (Lisman et al., 2007). Under previous assumptions neurotransmitter release activated the same subset of receptors regardless of its mode or timing, however, the dynamics of glutamate diffusion and AMPAR activation challenge this notion. The probability of AMPAR activation declines with distance from glutamate release due to both the low affinity of AMPARs for glutamate and the rapid diffusion of neurotransmitters following release (Franks et al., 2003; Raghavachari and Lisman, 2004). Modeling has predicted that the necessary concentration of glutamate to activate AMPARs following synaptic vesicle fusion is extremely brief and localized whereby presynaptic release most likely creates a “hotspot” of neurotransmitter activating only a subset of receptors (Biederer et al., 2017). Thus, now the prevailing view is both the location and timing of glutamate release is important for determining how information is transmitted at excitatory synapses.

### Plasticity

Synaptic plasticity involves both pre- and post-synaptic mechanisms that employ numerous molecules and signaling cascades. However, if we focus on receptor mediated postsynaptic potentiation we see how nano-organization at the synapse can be vital, where not just the number of postsynaptic receptors, but also receptor location is a critical determinant of synaptic strength and efficacy. For instance, during LTP induction, it has been reported that preventing AMPAR surface diffusion markedly impairs potentiation *in vitro* in addition to inhibiting behavioral aspects of contextual learning (Penn et al., 2017). Modeling predicts that increasing receptor density is more efficient than merely increasing synaptic area as the same degree of AMPAR current potentiation can be achieved by reducing inter-receptor distances by 30–35% or by increasing AMPAR number by 100–200% (Franks et al., 2003; Savtchenko and Rusakov, 2014).

**FIGURE 2:**
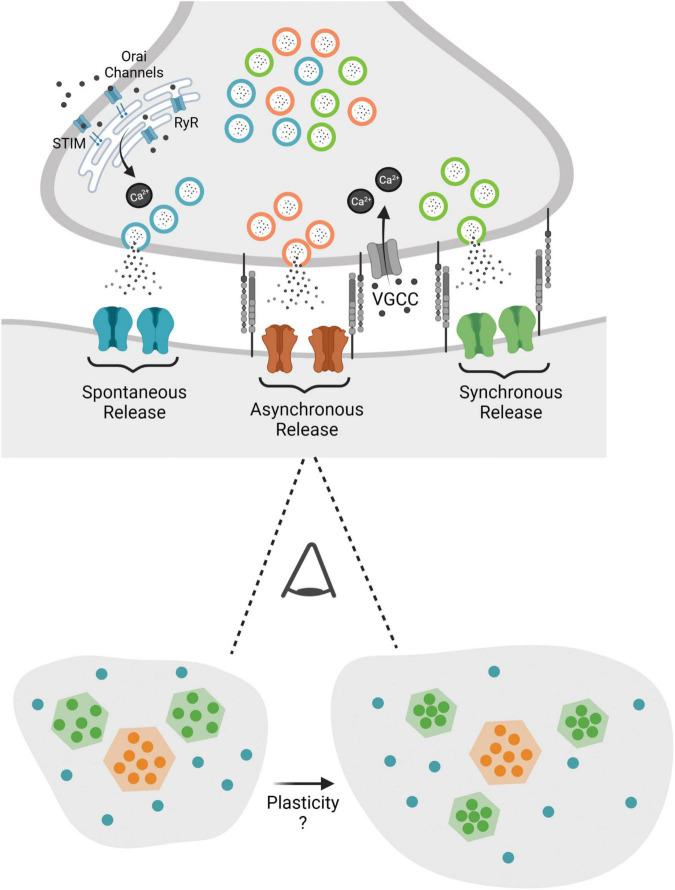
This figure depicts the nanostructure that is proposed to privilege different modes of release at the synapse, with evoked release supported by specific structural elements (i.e., neurexin and LRRTM2). When viewing the synapse from above one proposal states that evoked release is clustered in confined areas, while asynchronous release is clustered toward the center and spontaneous release is distributed over a larger area of the synapse. Potential plasticity processes may alter nanostructure with increased spine size and a parallel increase in nano-modules as well as increased postsynaptic scaffold and receptor density within nanocolumns.

However, not until the advent of super resolution microscopy has the nanoscale organization of a synapse been explicitly linked to synaptic strength. With super resolution microscopy these earlier proposals were validated with the visualization of intra-synapse AMPAR clusters. Chemical LTP induction lead to nano-domain alignment alterations of increased postsynaptic density protein 95 (PSD-95) density followed by trans-synaptic re-alignment within nanoclusters (Tang et al., 2016). In addition, structural plasticity, changes in spine and bouton size in response to activity paradigms, is related to synaptic molecular architecture. In that synaptic reorganization is modular, whereby increases in spine size are accompanied by the addition of individual nano-modules (Hruska et al., 2018; Figure 2). These studies collectively posit that purely re-distribution of receptors can alter synaptic strength and this reorganization can be a more resource effective means for postsynaptic potentiation.

## Molecular Components That Mediate Nano-Organization

As discussed above, all different modes of release have distinct functional roles, specific synaptic vesicle fusion machinery, and target distinct postsynaptic receptors, but how this organization is maintained structurally in a single active zone synapse remains poorly understood. The advancement of super resolution light microscopy techniques in conjunction with cryogenic electron microscopy (EM) approaches have uncovered prominent nano-organization at single synapses supported by molecular nano-scaffolds that link pre- and post-synaptic compartments providing a platform that privileges different regions of the synapse for evoked release.

Early optical studies hinted at synaptic nano-organization while examining clustering dynamics of receptor subpopulations. Relative to the extrasynaptic space, AMPARs are organized in nanodomains where the number of clusters increases with synapse size (MacGillavry et al., 2013; Biederer et al., 2017). These clusters are mobile and dynamic as AMPARs can diffuse in and out although they demonstrate an overall stability creating a dynamic environment of AMPAR localization (Nair et al., 2013). Therefore, it has become evident that the distribution of receptors in the excitatory postsynaptic density is not homogeneous whereby AMPARs and NMDARs are both arranged in clusters (Goncalves et al., 2020). Moreover, these receptor clusters are found in areas enriched in postsynaptic proteins providing a proteomic network supporting nanoscale receptor organization (MacGillavry et al., 2013). A principal protein of interest is PSD-95 due to both its role in anchoring AMPARs via TARP binding and its co-enrichment with AMPAR clusters at the postsynaptic density (PSD) (Chen et al., 2011; MacGillavry et al., 2013; Nair et al., 2013; Tang et al., 2016). Consequently, PSD-95 is thought to be the master organizer of excitatory postsynaptic organization facilitating the heterogeneous enrichment of receptors and other scaffolding proteins such as GKAP, Shank, and Homer, creating a range of protein expression within the postsynaptic neuron (Tang et al., 2016; Biederer et al., 2017).

A recent study demonstrated that presynaptic loci with a high density of proteins vital for vesicle priming and fusion (such as RIM1/2, Munc13, and bassoon) were associated with parallel postsynaptic gradients of PSD-95 and AMPARs (Tang et al., 2016). Here, presynaptic RIM1/2 clusters show the largest degree of correspondence with postsynaptic PSD-95 whereas presynaptic Munc13 and Bassoon show weaker association with their postsynaptic scaffold counterparts. These findings establish a trans-synaptic nanocolumn model via co-alignment and enrichment of both pre- and post-synaptic proteomes. This nanocolumn organization facilities concentrated increases in neurotransmitter at designated points along the active zone aligned with specific receptor subpopulations providing the substrate for the functionality of different types of release.

## Correspondence Between Nanostructure and Function

The organization of the synapse in nanocolumns deconstructs the classical homogeneous view of single active zone synapses and proposes a heterogeneous molecular platform that favors different modes of release at different regions governing distinct downstream biochemical signaling pathways. A substructure within the active zone that is privileged for evoked release was defined by the co-enrichment of pre- and post-synaptic proteins defined by high local RIM1, PSD-95, and GluA2 density. The mapping of evoked and spontaneous synaptic vesicle fusion in relation to the molecular layout of these key proteins demonstrated evoked fusion is privileged at areas of dense postsynaptic ensembles and spontaneous release happens within a greater area of the active zone (Figure 2). Active zone regions with the highest likelihood of release are aligned to the densest AMPAR areas, optimizing the potency of neurotransmission (Tang et al., 2016). This work demonstrated AMPARs are enriched within 80 nm nanodomains aligned with presynaptic RIM nanodomains, however, how AMPAR clusters opposed to release sites are maintained across the synapse was originally unclear.

The extracellular cleavage of leucine rich repeat transmembrane 2 (LRRTM2), a cell adhesion molecule that binds postsynaptic PSD-95 and presynaptic neurexin, has recently been demonstrated to be vital in regulating this pre- and post-synaptic alignment. The acute proteolytic cleavage LRRTM2, disrupting extracellular neurexin binding, led to the rapid repositioning of AMPARs away from RIM nanodomains, specifically reducing the amplitude of evoked PSCs while spontaneous release was unaffected. The enrichment of LRRTM2 in the nanocolumn upholds release and receptor nanoclustering by facilitating the alignment of AMPARs for evoked release (Ramsey et al., 2021). Ultimately demonstrating LRRTM2 as a structural link mediating the trans-synaptic alignment necessary for evoked neurotransmitter release (Figure 2). The bias of these defined nanocolumns toward one mode of release bolsters the premise that different modes of release activate different postsynaptic receptors and provides a substrate for plasticity. However, multiple questions still remain. First of all, synaptic nanostructure has been shown to facilitate evoked release, however, it is unknown if there are molecular platforms in place to support spontaneous release. Furthermore, are there other trans-synaptic molecules enriched in nanocolumns? If so, what is their role in development? In addition, are there other mechanisms besides direct interactions supporting pre- and post-synaptic domains, i.e., steric hinderance (Biederer et al., 2017) that impact nano-organization? Lastly how does this synaptic nanostructure relate to synaptic vesicle pools? As discussed above synaptic vesicle pools are defined by their molecular identity but how do nanodomains traffic and facilitate release at distinct fusions sites of molecularly diverse synaptic vesicles?

Recent development of a system that allowed for stimulation followed by the immediate high-pressure freezing of the sample, “zap-and-freeze,” allowed for the spatial and temporal analysis of both synchronous and asynchronous release at the active zone. Series of electron micrographs of synaptic ultrastructure revealed a distinct spatial organization of vesicle fusion following stimulation, with synchronous release happening throughout the active zone and asynchronous release, vesicles that fuse 11 ms after stimulation, concentrated at the center (Figure 2; Kusick et al., 2020). The functional significance of this segregation of action potential induced release was investigated in regard to subtypes of glutamate receptors that have a heterogeneous organization on the post synapse.

Excitatory synapses typically express three subtypes of glutamate receptors; NMDARs, AMPARs, and metabotropic glutamate receptors that display a centralized, peripheral, and dispersed organization within the PSD, respectively (Goncalves et al., 2020). The location of NMDARs is of particular interest due to the necessity of not only glutamate binding but postsynaptic depolarization, to relieve magnesium pore blockade, for receptor activation (Seeburg et al., 1995). The ultrastructural analysis of synaptic fusion pits and postsynaptic receptors delineated AMPARs cluster at the periphery and NMDARs at the center of the PSD. Further computer modeling revealed the spatial organization of fusion sites with specific receptors, where asynchronous release is privileged adjacent to where NMDARs are localized (Li et al., 2021). The temporal difference of synchronous and asynchronous release is suspected to facilitate NMDAR activation. Thus, providing new evidence deviating from the canonical view of NMDAR activation via spike timing dependent plasticity but NMDAR activation in the same active zone by the same spike due to the trans-synaptic alignment of release sites and receptors (Li et al., 2021).

These studies outline a synaptic organization of nanocolumn clusters supporting evoked release where asynchronous release is preferentially localized to the center of the synapse and spontaneous release happening throughout the synapse (Figure 2). Modular nanocolumn synaptic organization implies a design principle that may ultimately mediate the segregation of release, shape synaptic efficacy, determine neurotransmission reliability, and tune plasticity.

## Future Directions: Technology and Tools

The largest challenge with understanding the ultrastructure of the synapse in relation to neurotransmission is synaptic structures are too small for traditional light microscopy methods while physiological processes are fast and dynamic (Biederer et al., 2017). Use-dependent pharmacology to investigate the segregation of release primarily at excitatory hippocampal synapses have set the stage for advanced techniques to uncover synaptic nanostructure. However, these fundamental techniques should not be overlooked but employed to study inhibitory synapses as well as other synapse types to form a complete picture of synaptic organization throughout the brain.

The advent of super resolution microscopy has allowed us to overcome the diffraction limit of light and probe the nanoscale organization of the synapse with fluorescence microscopy. In addition, cryogenic electron microscopy (cryo-EM) techniques allow for the visualization of molecules providing great insight into the morphology of cells and confirmations of proteins. However, at this point cryo-EM does not endow the molecular specificity that fluorescence microscopy offers, and super resolution microscopy does not provide high resolution characterization of synaptic ultra-structure. Whilst super resolution microscopy and cryo-EM are great imaging tools, they do not provide the temporal resolution needed to see physiological synaptic processes in real time. Therefore, electrophysiology still has paramount importance and utility today due to its unparalleled temporal resolution, making it a technique that should be employed in addition to imaging to understand fundamental organizing principles of the synapse. An approach that has recently linked synapse nanostructure to function is cryo-EM visualization of synaptic vesicle fusion coined “zap and freeze” (Kusick et al., 2020). One of the largest draw backs of EM is it cannot be conducted on live tissue. Nevertheless, this method has been used to characterize synaptic ultrastructure directly following stimulation by reconstructing a series of events from snapshots (Kusick et al., 2020). However, it remains a challenge to visualize spontaneous release using these approaches as these fusion events are not under the experimenter’s control.

In order to fully elucidate the function of synaptic nanoscale organization, methods that provide molecular information as well as the monitoring of synaptic vesicle release and retrieval must be used in parallel. Nanometer level resolution has changed the field of neuroscience giving us the tools to further study how synaptic nanostructure underlies function, however, novel tools and further insight are necessary to causally link these nano-structural elements to synaptic signaling.

## Author Contributions

NG and EK wrote and edited this article. Both authors contributed to the article and approved the submitted version.

## Conflict of Interest

The authors declare that the research was conducted in the absence of any commercial or financial relationships that could be construed as a potential conflict of interest.

## Publisher’s Note

All claims expressed in this article are solely those of the authors and do not necessarily represent those of their affiliated organizations, or those of the publisher, the editors and the reviewers. Any product that may be evaluated in this article, or claim that may be made by its manufacturer, is not guaranteed or endorsed by the publisher.

## References

[B1] Alabi A. A. Tsien R. W. (2012). Synaptic vesicle pools and dynamics. *Cold Spring Harb. Perspect. Biol.* 4:a013680. 10.1101/cshperspect.a013680 22745285 PMC3405865

[B2] Alten B. Zhou Q. Shin O.-H. Esquivies L. Lin P.-Y. White K. I. (2021). Role of aberrant spontaneous neurotransmission in SNAP25-associated encephalopathies. *Neuron* 109 59–72.e5. 10.1016/j.neuron.2020.10.012 33147442 PMC7790958

[B3] Andreae L. C. Burrone J. (2015). Spontaneous neurotransmitter release shapes dendritic arbors via long-range activation of NMDA receptors. *Cell Rep.* 10 873–882. 10.1016/j.celrep.2015.01.032 25683710 PMC4542315

[B4] Andreae L. C. Burrone J. (2018). The role of spontaneous neurotransmission in synapse and circuit development. *J. Neurosci. Res.* 96 354–359. 10.1002/jnr.24154 29034487 PMC5813191

[B5] Andreae L. C. Fredj N. B. Burrone J. (2012). Independent vesicle pools underlie different modes of release during neuronal development. *J. Neurosci.* 32 1867–1874. 10.1523/JNEUROSCI.5181-11.2012 22302825 PMC6703344

[B6] Aoto J. Nam C. I. Poon M. M. Ting P. Chen L. (2008). Synaptic signaling by all-trans retinoic acid in homeostatic synaptic plasticity. *Neuron* 60 308–320. 10.1016/j.neuron.2008.08.012 18957222 PMC2634746

[B7] Atasoy D. Ertunc M. Moulder K. L. Blackwell J. Chung C. Su J. (2008). Spontaneous and evoked glutamate release activates two populations of NMDA receptors with limited overlap. *J. Neurosci.* 28 10151–10166. 10.1523/JNEUROSCI.2432-08.2008 18829973 PMC2578837

[B8] Autry A. E. Adachi M. Nosyreva E. Na E. S. Los M. F. Cheng P. (2011). NMDA receptor blockade at rest triggers rapid behavioural antidepressant responses. *Nature* 475 91–95. 10.1038/nature10130 21677641 PMC3172695

[B9] Bacaj T. Wu D. Yang X. Morishita W. Zhou P. Xu W. (2013). Synaptotagmin-1 and synaptotagmin-7 trigger synchronous and asynchronous phases of neurotransmitter release. *Neuron* 80 947–959. 10.1016/j.neuron.2013.10.026 24267651 PMC3888870

[B10] Bal M. Leitz J. Reese A. L. Ramirez D. M. O. Durakoglugil M. Herz J. (2013). Reelin mobilizes a VAMP7-dependent synaptic vesicle pool and selectively augments spontaneous neurotransmission. *Neuron* 80 934–946. 10.1016/j.neuron.2013.08.024 24210904 PMC3840105

[B11] Banerjee S. Vernon S. Jiao W. Choi B. J. Ruchti E. Asadzadeh J. (2021). Miniature neurotransmission is required to maintain Drosophila synaptic structures during ageing. *Nat. Commun.* 12:4399. 10.1038/s41467-021-24490-1 34285221 PMC8292383

[B12] Ben-Ari Y. (2002). Excitatory actions of gaba during development: the nature of the nurture. *Nat. Rev. Neurosci.* 3 728–739. 10.1038/nrn920 12209121

[B13] Biederer T. Kaeser P. S. Blanpied T. A. (2017). Trans-cellular nano-alignment of synaptic function. *Neuron* 96 680–696. 10.1016/j.neuron.2017.10.006 29096080 PMC5777221

[B14] Chanaday N. L. Kavalali E. T. (2017). How do you recognize and reconstitute a synaptic vesicle after fusion? *F1000Res* 6:1734. 10.12688/f1000research.12072.1 29034086 PMC5615776

[B15] Chanaday N. L. Kavalali E. T. (2018). Presynaptic origins of distinct modes of neurotransmitter release. *Curr. Opin. Neurobiol.* 51 119–126. 10.1016/j.conb.2018.03.005 29597140 PMC6066415

[B16] Chen J.-C. Lo Y.-F. Lin Y.-W. Lin S.-H. Huang C.-L. Cheng C.-J. (2019). WNK4 kinase is a physiological intracellular chloride sensor. *Proc. Natl. Acad. Sci. U.S.A* 116 4502–4507. 10.1073/pnas.1817220116 30765526 PMC6410802

[B17] Chen X. Nelson C. D. Li X. Winters C. A. Azzam R. Sousa A. A. (2011). PSD-95 is required to sustain the molecular organization of the postsynaptic density. *J. Neurosci.* 31 6329–6338. 10.1523/JNEUROSCI.5968-10.2011 21525273 PMC3099547

[B18] Choi B. J. Imlach W. L. Jiao W. Wolfram V. Wu Y. Grbic M. (2014). Miniature neurotransmission regulates Drosophila synaptic structural maturation. *Neuron* 82 618–634. 10.1016/j.neuron.2014.03.012 24811381 PMC4022839

[B19] Courtney N. A. Briguglio J. S. Bradberry M. M. Greer C. Chapman E. R. (2018). Excitatory and inhibitory neurons utilize different Ca^2 +^ sensors and sources to regulate spontaneous release. *Neuron* 98 977–991.e5. 10.1016/j.neuron.2018.04.022 29754754 PMC6090561

[B20] Crawford D. C. Kavalali E. T. (2015). Molecular underpinnings of synaptic vesicle pool heterogeneity. *Traffic* 16 338–364. 10.1111/tra.12262 25620674 PMC4578802

[B21] Crawford D. C. Ramirez D. M. O. Trauterman B. Monteggia L. M. Kavalali E. T. (2017). Selective molecular impairment of spontaneous neurotransmission modulates synaptic efficacy. *Nat. Commun*, 8:14436. 10.1038/ncomms14436 28186166 PMC5311059

[B22] Deák F. Schoch S. Liu X. Südhof T. C. Kavalali E. T. (2004). Synaptobrevin is essential for fast synaptic-vesicle endocytosis. *Nat. Cell Biol.* 6 1102–1108. 10.1038/ncb1185 15475946

[B23] Fatt P. Katz B. (1950). Some observations on biological noise. *Nature* 166, 597–598. 10.1038/166597a0 14780165

[B24] Franks K. M. Stevens C. F. Sejnowski T. J. (2003). Independent sources of quantal variability at single glutamatergic synapses. *J. Neurosci.* 23 3186–3195. 10.1523/JNEUROSCI.23-08-03186.2003 12716926 PMC2944019

[B25] Geppert M. Goda Y. Hammer R. E. Li C. Rosahl T. W. Stevens C. F. (1994). Synaptotagmin I: a major Ca2+ sensor for transmitter release at a central synapse. *Cell* 79 717–727. 10.1016/0092-8674(94)90556-8 7954835

[B26] Goncalves J. Bartol T. M. Camus C. Levet F. Menegolla A. P. Sejnowski T. J. (2020). Nanoscale co-organization and coactivation of AMPAR, NMDAR, and mGluR at excitatory synapses. *Proc. Natl. Acad. Sci. U.S.A.* 117 14503–14511. 10.1073/pnas.1922563117 32513712 PMC7321977

[B27] Gonzalez-Islas C. Bülow P. Wenner P. (2018). Regulation of synaptic scaling by action potential-independent miniature neurotransmission. *J. Neurosci. Res.* 96 348–353. 10.1002/jnr.24138 28782263 PMC5766397

[B28] Groffen A. J. Martens S. Arazola R. D. Cornelisse L. N. Lozovaya N. de Jong A. P. H. (2010). Doc2b is a high-affinity Ca^2 +^ sensor for spontaneous neurotransmitter release. *Science* 327 1614–1618. 10.1126/science.1183765 20150444 PMC2846320

[B29] Hefft S. Jonas P. (2005). Asynchronous GABA release generates long-lasting inhibition at a hippocampal interneuron-principal neuron synapse. *Nat. Neurosci.* 8 1319–1328. 10.1038/nn1542 16158066

[B30] Heubl M. Zhang J. Pressey J. C. Al Awabdh S. Renner M. Gomez-Castro F. (2017). GABA A receptor dependent synaptic inhibition rapidly tunes KCC2 activity via the Cl - -sensitive WNK1 kinase. *Nat. Commun.* 8:1776. 10.1038/s41467-017-01749-0 29176664 PMC5701213

[B31] Horvath P. M. Chanaday N. L. Alten B. Kavalali E. T. Monteggia L. M. (2021). A subthreshold synaptic mechanism regulating BDNF expression and resting synaptic strength. *Cell Rep.* 36:109467. 10.1016/j.celrep.2021.109467 34348149 PMC8371576

[B32] Horvath P. M. Piazza M. K. Monteggia L. M. Kavalali E. T. (2020). Spontaneous and evoked neurotransmission are partially segregated at inhibitory synapses. *Elife* 9:e52852. 10.7554/eLife.52852 32401197 PMC7250572

[B33] Hruska M. Henderson N. Le Marchand S. J. Jafri H. Dalva M. B. (2018). Synaptic nanomodules underlie the organization and plasticity of spine synapses. *Nat. Neurosci.* 21 671–682. 10.1038/s41593-018-0138-9 29686261 PMC5920789

[B34] Hua Z. Leal-Ortiz S. Foss S. M. Waites C. L. Garner C. C. Voglmaier S. M. (2011). v-SNARE composition distinguishes synaptic vesicle pools. *Neuron* 71 474–487. 10.1016/j.neuron.2011.06.010 21835344 PMC3155686

[B35] Huntwork S. Littleton J. T. (2007). A complexin fusion clamp regulates spontaneous neurotransmitter release and synaptic growth. *Nat. Neurosci.* 10 1235–1237. 10.1038/nn1980 17873870

[B36] Jackman S. L. Turecek J. Belinsky J. E. Regehr W. G. (2016). The calcium sensor synaptotagmin 7 is required for synaptic facilitation. *Nature* 529 88–91. 10.1038/nature16507 26738595 PMC4729191

[B37] Katz B. (1969). *The Release of Neural Transmitter Substances.* Liverpool: Liverpool University Press.

[B38] Kavalali E. T. (2015). The mechanisms and functions of spontaneous neurotransmitter release. *Nat. Rev. Neurosci.* 16, 5–16. 10.1038/nrn3875 25524119

[B39] Kavalali E. T. (2018). Spontaneous neurotransmission: a form of neural communication comes of age. *J. Neurosci. Res.* 96 331–334. 10.1002/jnr.24207 29219198 PMC5766395

[B40] Kavalali E. T. (2020). Neuronal Ca2+ signalling at rest and during spontaneous neurotransmission. *J. Physiol.* 598 1649–1654. 10.1113/JP276541 30735245

[B41] Kavalali E. T. Monteggia L. M. (2020). Targeting homeostatic synaptic plasticity for treatment of mood disorders. *Neuron* 106 715–726. 10.1016/j.neuron.2020.05.015 32497508 PMC7517590

[B42] Klausberger T. Somogyi P. (2008). Neuronal Diversity and temporal dynamics: the unity of hippocampal circuit operations. *Science* 321 53–57. 10.1126/science.1149381 18599766 PMC4487503

[B43] Kononenko N. L. Haucke V. (2015). Molecular mechanisms of presynaptic membrane retrieval and synaptic vesicle reformation. *Neuron* 85 484–496. 10.1016/j.neuron.2014.12.016 25654254

[B44] Kusick G. F. Chin M. Raychaudhuri S. Lippmann K. Adula K. P. Hujber E. J. (2020). Synaptic vesicles transiently dock to refill release sites. *Nat. Neurosci.* 23 1329–1338. 10.1038/s41593-020-00716-1 32989294 PMC8054220

[B45] Li S. Raychaudhuri S. Lee S. A. Brockmann M. M. Wang J. Kusick G. (2021). Asynchronous release sites align with NMDA receptors in mouse hippocampal synapses. *Nat. Commun.* 12:677. 10.1038/s41467-021-21004-x 33514725 PMC7846561

[B46] Li Y. C. Chanaday N. L. Xu W. Kavalali E. T. (2017). Synaptotagmin-1- and synaptotagmin-7-dependent fusion mechanisms target synaptic vesicles to kinetically distinct endocytic pathways. *Neuron* 93 616–631.e3. 10.1016/j.neuron.2016.12.010 28111077 PMC5300960

[B47] Lin P.-Y. Chanaday N. L. Horvath P. M. Ramirez D. M. O. Monteggia L. M. Kavalali E. T. (2020). VAMP4 maintains a Ca2+-sensitive pool of spontaneously recycling synaptic vesicles. *J. Neurosci.* 40 5389–5401. 10.1523/JNEUROSCI.2386-19.2020 32532887 PMC7343330

[B48] Lin P.-Y. Kavalali E. T. Monteggia L. M. (2018). Genetic dissection of presynaptic and postsynaptic BDNF-TrkB signaling in synaptic efficacy of CA3-CA1 synapses. *Cell Rep.* 24 1550–1561. 10.1016/j.celrep.2018.07.020 30089265 PMC7176480

[B49] Lisman J. E. Raghavachari S. Tsien R. W. (2007). The sequence of events that underlie quantal transmission at central glutamatergic synapses. *Nat. Rev. Neurosci.* 8 597–609. 10.1038/nrn2191 17637801

[B50] Liu H. Dean C. Arthur C. P. Dong M. Chapman E. R. (2009). Autapses and networks of hippocampal neurons exhibit distinct synaptic transmission phenotypes in the absence of synaptotagmin I. *J. Neurosci.* 29 7395–7403. 10.1523/JNEUROSCI.1341-09.2009 19515907 PMC2723061

[B51] Luo F. Südhof T. C. (2017). Synaptotagmin-7-mediated asynchronous release boosts high-fidelity synchronous transmission at a central synapse. *Neuron* 94 826–839.e3. 10.1016/j.neuron.2017.04.020 28521135

[B52] MacGillavry H. D. Song Y. Raghavachari S. Blanpied T. A. (2013). Nanoscale scaffolding domains within the postsynaptic density concentrate synaptic AMPA receptors. *Neuron* 78 615–622. 10.1016/j.neuron.2013.03.009 23719161 PMC3668352

[B53] Maximov A. Südhof T. C. (2005). Autonomous function of synaptotagmin 1 in triggering synchronous release independent of asynchronous release. *Neuron* 48 547–554. 10.1016/j.neuron.2005.09.006 16301172

[B54] Melom J. E. Akbergenova Y. Gavornik J. P. Littleton J. T. (2013). Spontaneous and evoked release are independently regulated at individual active zones. *J. Neurosci.* 33 17253–17263. 10.1523/JNEUROSCI.3334-13.2013 24174659 PMC3812501

[B55] Molnár Z. López-Bendito G. Small J. Partridge L. D. Blakemore C. Wilson M. C. (2002). Normal development of embryonic thalamocortical connectivity in the absence of evoked synaptic activity. *J. Neurosci.* 22 10313–10323. 10.1523/JNEUROSCI.22-23-10313.2002 12451131 PMC6758728

[B56] Mori Y. Takenaka K. Fukazawa Y. Takamori S. (2021). The endosomal Q-SNARE, Syntaxin 7, defines a rapidly replenishing synaptic vesicle recycling pool in hippocampal neurons. *Commun. Biol.* 4:981. 10.1038/s42003-021-02512-4 34408265 PMC8373932

[B57] Nair D. Hosy E. Petersen J. D. Constals A. Giannone G. Choquet D. (2013). Super-resolution imaging reveals that AMPA receptors inside synapses are dynamically organized in nanodomains regulated by PSD95. *J. Neurosci.* 33 13204–13224. 10.1523/JNEUROSCI.2381-12.2013 23926273 PMC6619720

[B58] Pang Z. P. Bacaj T. Yang X. Zhou P. Xu W. Südhof T. C. (2011). Doc2 supports spontaneous synaptic transmission by a Ca2+-independent mechanism. *Neuron* 70 244–251. 10.1016/j.neuron.2011.03.011 21521611 PMC3102832

[B59] Peled E. S. Newman Z. L. Isacoff E. Y. (2014). Evoked and spontaneous transmission favored by distinct sets of synapses. *Curr. Biol.* 24 484–493. 10.1016/j.cub.2014.01.022 24560571 PMC4017949

[B60] Penn A. C. Zhang C. L. Georges F. Royer L. Breillat C. Hosy E. (2017). Hippocampal LTP and contextual learning require surface diffusion of AMPA receptors. *Nature* 549 384–388. 10.1038/nature23658 28902836 PMC5683353

[B61] Piala A. T. Moon T. M. Akella R. He H. Cobb M. H. Goldsmith E. J. (2014). Chloride sensing by WNK1 involves inhibition of autophosphorylation. *Sci. Signal.* 7:ra41. 10.1126/scisignal.2005050 24803536 PMC4123527

[B62] Raghavachari S. Lisman J. E. (2004). Properties of quantal transmission at CA1 synapses. *J. Neurophysiol.* 92 2456–2467. 10.1152/jn.00258.2004 15115789

[B63] Raingo J. Khvotchev M. Liu P. Darios F. Li Y. C. Ramirez D. M. O. (2012). VAMP4 directs synaptic vesicles to a pool that selectively maintains asynchronous neurotransmission. *Nat. Neurosci.* 15 738–745. 10.1038/nn.3067 22406549 PMC3337975

[B64] Ramirez D. M. O. Crawford D. C. Chanaday N. L. Trauterman B. Monteggia L. M. Kavalali E. T. (2017). Loss of Doc2-dependent spontaneous neurotransmission augments glutamatergic synaptic strength. *J. Neurosci.* 37 6224–6230. 10.1523/JNEUROSCI.0418-17.2017 28539418 PMC5490061

[B65] Ramirez D. M. O. Khvotchev M. Trauterman B. Kavalali E. T. (2012). Vti1a identifies a vesicle pool that preferentially recycles at rest and maintains spontaneous neurotransmission. *Neuron* 73 121–134. 10.1016/j.neuron.2011.10.034 22243751 PMC3259527

[B66] Ramsey A. M. Tang A.-H. LeGates T. A. Gou X.-Z. Carbone B. E. Thompson S. M. (2021). Subsynaptic positioning of AMPARs by LRRTM2 controls synaptic strength. *Sci. Adv.* 7:eabf3126. 10.1126/sciadv.abf3126 34417170 PMC8378824

[B67] Reese A. L. Kavalali E. T. (2015). Spontaneous neurotransmission signals through store-driven Ca(2+) transients to maintain synaptic homeostasis. *Elife* 4:e09262. 10.7554/eLife.09262 26208337 PMC4534843

[B68] Reese A. L. Kavalali E. T. (2016). Single synapse evaluation of the postsynaptic NMDA receptors targeted by evoked and spontaneous neurotransmission. *eLife* 5:e21170. 10.7554/eLife.21170 27882871 PMC5148599

[B69] Revelo N. H. Kamin D. Truckenbrodt S. Wong A. B. Reuter-Jessen K. Reisinger E. (2014). A new probe for super-resolution imaging of membranes elucidates trafficking pathways. *J. Cell Biol.* 205 591–606. 10.1083/jcb.201402066 24862576 PMC4033769

[B70] Rey S. A. Smith C. A. Fowler M. W. Crawford F. Burden J. J. Staras K. (2015). Ultrastructural and functional fate of recycled vesicles in hippocampal synapses. *Nat. Commun.* 6:8043. 10.1038/ncomms9043 26292808 PMC4560786

[B71] Rozov A. Bolshakov A. P. Valiullina-Rakhmatullina F. (2019). The ever-growing puzzle of asynchronous release. *Front. Cell. Neurosci.* 13:28. 10.3389/fncel.2019.00028 30809127 PMC6379310

[B72] Sara Y. Bal M. Adachi M. Monteggia L. M. Kavalali E. T. (2011). Use-dependent AMPA receptor block reveals segregation of spontaneous and evoked glutamatergic neurotransmission. *J. Neurosci.* 31 5378–5382. 10.1523/JNEUROSCI.5234-10.2011 21471372 PMC3086544

[B73] Savtchenko L. P. Rusakov D. A. (2014). Moderate AMPA receptor clustering on the nanoscale can efficiently potentiate synaptic current. *Philos. Trans. R. Soc. Lond. B Biol. Sci.* 369:20130167. 10.1098/rstb.2013.0167 24298165 PMC3843895

[B74] Schoch S. Deák F. Königstorfer A. Mozhayeva M. Sara Y. Südhof T. C. (2001). SNARE function analyzed in synaptobrevin/VAMP knockout mice. *Science* 294 1117–1122. 10.1126/science.1064335 11691998

[B75] Seeburg P. H. Burnashev N. Köhr G. Kuner T. Sprengel R. Monyer H. (1995). The NMDA receptor channel: molecular design of a coincidence detector. *Recent Prog. Horm. Res.* 50 19–34. 10.1016/b978-0-12-571150-0.50006-8 7740157

[B76] Südhof T. C. (2013). Neurotransmitter release: the last millisecond in the life of a synaptic vesicle. *Neuron* 80 675–690. 10.1016/j.neuron.2013.10.022 24183019 PMC3866025

[B77] Südhof T. C. Rothman J. E. (2009). Membrane fusion: grappling with SNARE and SM proteins. *Science* 323 474–477. 10.1126/science.1161748 19164740 PMC3736821

[B78] Sugita S. Shin O.-H. Han W. Lao Y. Südhof T. C. (2002). Synaptotagmins form a hierarchy of exocytotic Ca(2+) sensors with distinct Ca(2+) affinities. *EMBO J.* 21 270–280. 10.1093/emboj/21.3.270 11823420 PMC125835

[B79] Sutton M. A. Ito H. T. Cressy P. Kempf C. Woo J. C. Schuman E. M. (2006). Miniature neurotransmission stabilizes synaptic function via tonic suppression of local dendritic protein synthesis. *Cell* 125 785–799. 10.1016/j.cell.2006.03.040 16713568

[B80] Takamori S. Holt M. Stenius K. Lemke E. A. Grønborg M. Riedel D. (2006). Molecular anatomy of a trafficking organelle. *Cell* 127 831–846. 10.1016/j.cell.2006.10.030 17110340

[B81] Tang A.-H. Chen H. Li T. P. Metzbower S. R. MacGillavry H. D. Blanpied T. A. (2016). A trans-synaptic nanocolumn aligns neurotransmitter release to receptors. *Nature* 536 210–214. 10.1038/nature19058 27462810 PMC5002394

[B82] Turrigiano G. G. Nelson S. B. (2004). Homeostatic plasticity in the developing nervous system. *Nat. Rev. Neurosci.* 5 97–107. 10.1038/nrn1327 14735113

[B83] Walter A. M. Kurps J. de Wit H. Schöning S. Toft-Bertelsen T. L. Lauks J. (2014). The SNARE protein vti1a functions in dense-core vesicle biogenesis. *EMBO J.* 33 1681–1697. 10.15252/embj.201387549 24902738 PMC4194101

[B84] Williams C. L. Smith S. M. (2018). Calcium dependence of spontaneous neurotransmitter release. *J. Neurosci. Res.* 96 335–347. 10.1002/jnr.24116 28699241 PMC5766384

[B85] Xu W. Morishita W. Buckmaster P. S. Pang Z. P. Malenka R. C. Südhof T. C. (2012). Distinct neuronal coding schemes in memory revealed by selective erasure of fast synchronous synaptic transmission. *Neuron* 73 990–1001. 10.1016/j.neuron.2011.12.036 22405208 PMC3319466

